# The impact of splenectomy on human lipid metabolism

**DOI:** 10.48101/ujms.v127.8500

**Published:** 2022-06-07

**Authors:** Orgun Gunes, Emre Turgut, Yusuf Murat Bag, Ersin Gundoğan, Ajda Gunes, Fatih Sumer

**Affiliations:** a Department of Gastrointestinal Surgery, Atatürk Training and Research Hospital, Izmir, Turkey; b Department of Gastrointestinal Surgery, Inonu University Turgut Ozal Medical Center, Malatya, Turkey; c Department of Gastrointestinal Surgery, Van Training and Research Hospital, Van, Turkey; d Department of Gastrointestinal Surgery, Kayseri Training and Research Hospital, Kayseri, Turkey; e Department of Hematology, Ege University, Izmir, Turkey; f Department of Gastrointestinal Surgery, Irmet Hospital, Tekirdağ, Turkey

**Keywords:** atherosclerosis, hyperlipidemia, autotransplantation, homeostasis, coronary, lipoprotein

## Abstract

**Background:**

Splenectomy impacts hematological, immunological, and metabolic functions of the patient. Since our understanding of its metabolic effects, in particular effects on lipid metabolism, is limited, this study aims to investigate the effects of splenectomy on lipid metabolism.

**Methods:**

The data from 316 patients undergoing splenectomy between 2009 and 2019 were retrospectively analyzed. Thirty-eight patients whose serum lipid values were measured both preoperatively and 1 year after surgery were included in this study.

**Results:**

Significantly higher levels of total cholesterol, low-density lipoprotein (LDL), and non-high-density lipoprotein (HDL) lipid profile were found in the postsplenectomy measurements. However, no significant differences were recorded in levels of triglyceride, HDL, or very-LDL.

**Conclusion:**

We determined that splenectomy does impact lipid metabolism, and that the metabolic effects of splenectomy should further be investigated.

## Introduction

As part of the reticuloendothelial system, the spleen plays an important role in humoral and cellular immune response (1). Splenectomy used to be a common procedure for the treatment of a variety of diseases such as traumas, hematological diseases, and malignancies. However, as our understanding of the immune and hematological functions of the spleen has improved over the years, the indications for splenectomy have changed, and spleen-preserving surgical choices are now preferred (2). The metabolic effects of splenectomy, in particular, are still not fully understood, and its effects on lipid metabolism are a topic of current investigation. However, most studies on this topic that are reported in the literature are experimental and have produced differing and sometimes contradictory results. In this study, therefore, we aimed to investigate the effects of splenectomy on the lipid metabolism in our patients.

## Materials and methods

This study was approved by the institutional review board (approval no. 2021/1751). The data of 316 patients who had undergone splenectomy surgery between 2009 and 2019 were retrospectively analyzed. Patients using medication for lipid metabolism and those suffering from malnutrition were excluded from the study. A total of 38 patients whose blood lipid values had been recorded prior to splenectomy and 1 year after surgery were included in this study. The demographic data of the patients were analyzed, and pre- and post-surgery values for body mass index (BMI), total cholesterol (TC), low-density lipoprotein (LDL), high-density lipoprotein (HDL), non-HDL, very-low-density lipoprotein (VLDL), and triglyceride (TG) were compared. The serum samples were obtained following 12 h of fasting, and the measurements were done using the spectrophotometric method. Besides, preoperative and postoperative HDL and non-HDL values were evaluated also for two subgroups (splenectomies for autoimmune indications and splenectomies for non-autoimmune indications).

### Statistical analysis

Continuous variables were analyzed by paired sample’s t-test. Categorical variables were analyzed using the Chi-squared test and Fisher’s exact test. Statistical significance was taken as *P* < 0.05. The IBM SPSS Statistics for Windows, version 25.0 (IBM Corp., Armonk, NY, USA), was used for analysis.

## Results

Demographic features are given in Table 1. The mean age of the patient group was 44.4 years, with 27 patients (71%) being female. The most common indication for surgery was immune thrombocytopenic purpura. Comorbidities were detected in 13 (34%) patients. These comorbidities were primary hypertension in six patients, type-1 diabetes mellitus in three patients, allergic asthma in three patients, coronary artery disease in one patient, and Familial Mediterranean Fever in one patient. Twelve patients had one comorbidity, and one patient had two comorbidities. Perioperative mortality did not occur.

**Table 1:** Demographic data.

Variables	n(%)
Gender (Female/male)	27 (71%)/11 (29%)
Age	44.4 ± 16.0
Comorbidities	13 (34%)
Diagnosis	
Immune thrombocytopenic purpura	19 (50%)
Trauma	10 (26%)
Splenic abscess	3 (8%)
Splenic cyst	2 (5%)
Hereditary spherocytosis	2 (5%)
Autoimmune hemolytic anemia	1 (3%)
Hemangioma	1 (3%)

Table 2 shows levels of BMI, TC, VLDL, HDL, TG, and LDL in patients before and after splenectomy surgery. Although TC, LDL, and non-HDL values were found to be significantly higher after splenectomy, there was no significant change in BMI, TG, HDL, or VLDL. In addition, non-HDL values were found to be higher after splenectomy in both autoimmune indications and non-autoimmune indications. (Table 3)

**Table 2:** Preoperative and postoperative BMI (kg/m^2^) and lipid profile (mmol/L).

	Preoperative	Postoperative	*p*
BMI	26.4 ± 3.85	26.3 ± 4.16	0.91
TC	9.03 ± 2.50	10.37 ± 2.00	**0.01**
LDL	5.23 ± 1.76	6.10 ± 1.53	**0.02**
TG	7.52 ± 5.29	7.60 ± 5.45	0.95
VLDL	1.49 ± 1.06	1.52 ± 1.11	0.89
HDL	43.2 ± 16.6	49.0 ± 13.3	0.09
Non-HDL	6.92 ± 2.23	7.77 ± 2.15	**0.01**
Platelets*	137.5 ± 138.9	390.3 ± 176.5	**0.0001**
Hemoglobin**	12.3 ± 2.4	12.4 ± 2.5	0.86

BMI, body mass index; TC, total cholesterol; LDL, low-density lipoprotein; HDL, high-density lipoprotein; VLDL, very-low-density lipoprotein; TG, triglyceride.

* Units are expressed as × 10^9^/L.

** Units are expressed as g/dL.

Bold values indicate statistical significance (*p* < 0.05).

**Table 3:** Comparisons of preoperative and postoperative HDL and Non-HDL levels (mmol/l) of the autoimmune and non-autoimmune indications subgroups.

	Splenectomies for autoimmune indications (n=20)			Splenectomies for non-autoimmune indications (n=18)		
	Preoperative	Postoperative	P value	Preoperative	Postoperative	P value
HDL level	2.39± 0.93	2.64± 0.70	0.186	2.46± 0.94	2.80± 0.75	0.134
Non-HDL level	6.83± 2.29	7.57± 2.15	**0.002**	7.03± 2.22	7.99± 2.20	**0.010**

*Bold values indicate statistical significance (p < 0.05).”

## Discussion

As yet, there is no clear understanding of exactly how splenectomy impacts the immunological, hematological, and, in particular, the metabolic systems. The most severe known outcome is overwhelming post-splenectomy infection with a frequency of 0.1–3.2% and a mortality rate of 50% (3, 4). Another problematic effect is reactive thrombocytosis, which can be seen at a rate of up to 75% (5). Reactive thrombocytosis may cause venous thromboembolism, extensive intravascular coagulation, and, in the long term, endothelial damage and pulmonary hypertension (5, 6).

Meanwhile, research into the effects of splenectomy on lipid metabolism, which is thought to further escalate vascular complications, continues. There are several interpretations explaining the way in which splenectomy impacts lipid metabolism. The most popular of these, suggested by Schmidt et al., is that the spleen acts as a reservoir for lipids (7). According to another theory, macrophages store fat through phagocytosis. In the case of hypersplenism, hypolipidemia will develop due to the increase in macrophage phagocytosis and splenic volume. On the other hand, splenectomy will cause the opposite effect (8); by means of B lymphocytes, it increases the production of anti-oxLDL antibodies against oxidized LDL (oxLDL), which is thought to increase splenic atherosclerosis (9). It is also thought to be involved in LDL catabolism (10, 11).

The liver is the main site for lipid metabolism, and this is where Kupffer cells regulate lipid metabolism through hepatocytic phagocytosis and the expression of the enzyme lipoprotein lipase (LPL). According to another theory, the LPL activity has also been detected in the spleen, and splenectomy reduces the Kupffer cells’ regulatory ability (12–14). Although the mechanism is not fully understood, the changes in the postsplenectomy miRNA expression could establish a connection between immune-mediated mechanisms and atherosclerosis (15).

The increase in TG and LDL and decrease in HDL are known as the atherogenic lipid triad (16). Considering that atherosclerotic diseases are the most common cause of death, it is important to understand the effect of splenectomy on the lipid profile.

Most studies investigating the effect of splenectomy on the lipid profile and atherosclerosis are experimental and have provided contradictory results. In his study on dogs in 1914, King found that cholesterol levels increased after splenectomy (17). Asai et al. reported higher TG, TC, and phospholipid, lower HDL, and no change in LDL in their study on rabbits (18). Petroianu (19) and Aviram (20) worked with rats and noted increased TC and LDL and decreased HDL. Fatouros detected high TG and low HDL (21). In another study by Altinel et al., LDL, oxLDL, and TG were all found to be high (22). Meanwhile, in six other studies, there was no change at all in the lipid profiles (9, 23–27). However, atherosclerosis was seen more frequently in three of these studies (9, 23, 24), the incidence did not change in two of them (26, 27), and interestingly, atherosclerosis was found less frequently in the study conducted by Li et al. (25). Furthermore, Akan (12) and Lemos-Paulo (28) found that all lipid parameters increased after splenectomy, while Akan (12) found that their levels returned to normal with autotransplantation.

It is likely that splenectomy may impact atherosclerosis by affecting lipid homeostasis, the inflammatory processes, and coagulation. In a study conducted in the 1970s, Robinette and Fraumeni Jr. noticed high mortality due to cardiovascular disease in veterans, who had undergone splenectomy during World War II. The authors concluded that splenectomy may affect the lipid profile (10). In a more recent study, cardiovascular diseases were also found to be higher in veterans who had had splenectomy, although this difference disappeared when those with autoimmune disease were excluded from the splenectomy group (29). In this study, even if the number of patients was insufficient, HDL and non-HDL values were compared in subgroups with and without autoimmune indication.

To the best of our knowledge, the only study in the literature involving human patients is that of Aviram, which involves three patients and two control groups and evaluates the lipid profile for 4 days post-surgery. Unlike other research in the literature, ours is a retrospective study on humans and evaluates the long-term effects of splenectomy. In this study, we found elevated LDL, TC, and non-HDL levels, but no change in TG, VLDL, or HDL. When subgroups with and without autoimmune indication were evaluated, we found that non-HDL levels were higher after splenectomy in both groups.

## Limitations

This study has certain limitations. First, it is a retrospective study. Second, the incidence of patient atherosclerosis was not considered during the long-term follow-up. Third, the number of patients is relatively small.

## Conclusion

We found that splenectomy had a negative impact on the lipid metabolism of our patients. We believe that, after splenectomy surgery, the metabolic impact should be monitored, in addition to immune and hematological effects.
